# Supplementary material for: Are carbon emissions trading and green financial instruments synergistic? -Comprehensive quantitative research based on content analysis
Source: PLoS One. 2024 Mar 7;19(3):e0298601. doi: 10.1371/journal.pone.0298601 (PMC10919601; doi:10.1371/journal.pone.0298601)
Supplement: S1 Data — (ZIP) [file pone.0298601.s001.zip › Data/地级市Z_F工作报告.zip!/╔╜╢1⁄2/╚╒╒╒╩╨/└┤╫╘╣1⁄2╓┌║┼í╛┬φ┐╦╩2╛▌═°í┐_2004.docx]

各位代表：

现在，我代表市人民政府向大会作政府工作报告，请予审议，并请市政协委员和其他列席会议的同志提出意见。

2003年政府工作的回顾

刚刚过去的一年，是不平凡的一年，突如其来的非典疫情，给我们带来了严峻考验。全市人民在省委、省政府和市委的正确领导下，坚持以邓小平理论和“三个代表”重要思想为指导，紧紧围绕跨越发展的目标，解放思想，干事创业，加快发展，圆满完成了市十五届人大一次会议确定的各项任务，夺取了抗击非典与经济社会发展的双胜利，一些主要经济指标创历史最好水平，许多事关全局的工作取得重大突破，实现了跨越发展的良好开局。

一、经济发展跃上新台阶。发展速度明显加快。初步核算，2003年全市国内生产总值突破300亿元大关，达到311.67亿元，人均GDP首次超过1万元。经济增长率达到17%，创1996年以来最高水平。一、二、三产业增加值分别增长5.2%、24.4%和14.7%。规模以上工业增加值增长31%，居全省第三位；利税、利润分别增长43%和68.4%。工业用电量增长23.9%，居全省第三位。商贸流通繁荣活跃，社会消费品零售总额按可比口径增长14.7%。金融机构存贷款余额双双突破200亿元大关，分别增长24.5%和 27.5%。实现地方财政收入11.15亿元，按可比口径增长12.18%。产业结构明显优化。三次产业比例由上年的20.4：41.4：38.2调整为18.5：44.6：36.9，二、三产业比重首次突破80%。全市新增高效经济作物11万亩，从事二、三产业的农民占乡村从业人员的比重达到34.6%，销售收入过亿元的农业龙头企业发展到8家。规模以上工业增加值占全部工业增加值的比重达到55.7%，比上年提高4.6个百分点。非公有制经济所占比重达到48%，比上年提高2.5个百分点。发展后劲明显增强。完成全社会固定资产投资131.24亿元，增长54%，是1994年以来增幅最高的年份，其中工业投入43.9亿元，增长 106.3%。特别是一批大项目的引进建设，为经济发展增强了活力和后劲。

二、对外开放实现新跨越。开放环境和条件得到较大改善。在硬环境上，开通了日照至韩国平泽的客箱班轮航线，圆了日照人民多年的“通航梦”，架起了对韩经贸往来的“金桥”；建成了胶新铁路和同三高速公路日照段，强化了与半岛城市群的联系；拓展了日照、岚山经济开发区等园区的发展空间，载体功能明显增强。在软环境上，完成了第二轮行政审批制度改革，成立了城市管理行政执法局、行政服务中心和行政投诉监察中心，办事效率和服务质量明显提高。招商引资取得突破性进展。全市共招引合同以上项目1954个，实际到位资金86.3亿元，其中总投资1亿元至10亿元的35 个、10亿元以上的4个。合同利用外资2.21亿美元，实际利用外资1.33亿美元，分别增长119.5%和 137.7%。特别是在大规模利用外资上取得历史性突破，新加坡金鹰国际集团投资12亿美元建设年产 100万吨的木浆项目，创下了全省单个项目利用外资之最。对外贸易快速增长。完成进出口总值 12.91亿美元，增长39.8%，其中加工贸易增长47.4%，高于一般贸易35.6个百分点。经济外向度达到 34.3%，比上年提高5.1个百分点。

三、港口经济开创新局面。港口体制改革取得突破。顺利完成了两港联合重组，组建了日照港（集团）有限公司，实现了政企分开和一城一港、港城一体的目标，港口发展揭开了新的一页。港口建设步入加速期。矿石码头、集装箱码头等10项重点工程开工建设，日照港西港区通用泊位、国际候船厅投入使用，完成港口投资10.5亿元，是“九五”期间的近两倍。港口吞吐能力有了新提高。完成港口货物吞吐量4506.5万吨，集装箱运量9.62万标箱，分别增长18.4%和53.5%。海上国际客运实现历史性突破，出入境旅客达到1.95万人次。临港工业框架初步形成。围绕培育冶金、粮油加工、液体化工、木制品加工、浆纸、能源六大临港产业，开工建设投资过亿元的临港工业项目12个，完成投资21.4亿元。这些项目全部建成投产后，可新增工业增加值35亿元、销售收入108亿元、利税 13.5亿元。特别是总投资超过百亿元、年产500万吨的日照钢铁项目一期工程投产，圆了全市人民的 “钢铁梦”。

四、市场取向的改革迈出新步伐。去年是市场化运作力度最大、效果最好的一年。一是用市场经济的理念建立了土地要素市场。在政府高度垄断土地一级市场的前提下，对经营性用地全部实行招标、拍卖、挂牌出让，并将市场机制扩大到城中村改造、城区工业用地“退二进三”及科教文卫用地等领域，出台了一系列政策措施，既保证了土地市场的规范有序、公平公正，又保护了失地农民的合法利益，增加了政府收益。全年实现政府土地总收益11.87亿元，是上年的3.1倍，增幅居全省第一。二是用市场化的方式改革城市建管体制。对市环卫处的职能实行政企分开、管干分离，组建了16个专业经营公司，将道路保洁权公开招标拍卖，实现了由“花钱养人”到“花钱买干净”的转变；将80余座公厕的建设和经营权推向市场，对能实行招标的市政建设工程全部实行招标。三是用市场化的办法实施经济适用房政策，运作大型会展活动。对经济适用房实行了“用地市场化、审批程序化、补贴货币化、购房自主化、监管规范化”改革，实现了住房困难户、房地产开发商和政府三满意。首届茶博会、房博会、中韩投资贸易博览会、省农运会等经贸会展和体育赛事活动的举办，也都按市场化方式运作，政府没花一分钱。

五、发展思路有了新突破。经过积极争取，省委、省政府把日照纳入了半岛城市群，使我市跨入山东对外开放的第一方阵，给加快发展带来了历史性机遇。为抢抓机遇，乘势而上，我们立足“半岛城市”这一新的定位，科学分析市情，重新审视日照的发展，对事关全局的重大战略问题进行深入调研，进一步明确了发展思路和战略规划。一是确立了跨越发展的奋斗目标，积极开展学赶威海活动，做到了学有榜样、赶有标兵，使跨越发展的信心转化为具体的行动。二是确立了依托青岛发展日照的思路，出台了《关于接轨青岛融入半岛城市群的意见》，提出日照要加快发展，必须主动融入青岛经济圈，要在思想观念、产业发展、交通体系等方面积极与青岛接轨，提高主动接受青岛辐射带动的能力。三是把港口经济作为日照发展的重点，从港口建设、临港工业和港口物流业三个方面进行积极培育，提出把日照港建成亿吨大港，将岚山港北扩，为再建一个亿吨大港预留发展空间，进一步凸显了日照的港口优势。四是聘请中国城市规划设计院开展了城市空间发展战略研究，出台了《生态市建设规划》。这些思路和战略规划的调整，将对我市经济和社会发展起到重要的作用。

六、社会事业取得新进步。科教兴市战略深入实施。科技创新和技术推广服务体系建设取得明显成效，高新技术产业产值增长50.3%。中小学课程改革全面启动，改造危房43万平方米，高中段教育普及率提高16个百分点。特别是大学科技园建设取得新进展，新引进中国地质大学、西安翻译学院两所高校，进园大学达到7所，完成建筑面积80万平方米，在校大学生达到2万人。可持续发展成效明显。全面实施生态市建设规划，积极开展争创国家卫生城市和环保模范城市活动，大气质量全年保持优良，在国家环保总局7月份公布的全国环保重点城市环境空气质量排行榜中，日照名列第三。人口和计划生育工作连续六年被省委、省政府授予一等奖。海洋、国土资源保护工作进一步加强，耕地总量保持动态平衡。城市面貌发生明显变化。完成城市基础设施投资4.8亿元，建成了上海路、山海路等28条城市道路和奎山供水二期、“北水南调”、污水处理厂等一批重点工程，新增城市绿地 80万平方米，人均达到15平方米，被评为省级园林城市。经过全市上下的共同努力，我市成功地获得了“中国优秀旅游城市”的荣誉称号。精神文明和民主法制建设得到加强。积极推进城市文化建设，文化产业逐步发育。体育事业取得新进展，成功地承办了全国跆拳道锦标赛等6项省级以上赛事。积极开展双拥共建活动，实现了争创全国双拥模范城“二连冠”。坚持依法治市，加强普法教育，深入开展“严打”斗争，治安秩序保持稳定。妥善处理人民内部矛盾，群众来信来访和集访案件分别下降8.6%和38.2%。强化安全生产监督管理，安全事故发生起数和死亡人数分别下降 28.3%和7.9%。新闻出版、民族宗教、外事侨务、对台事务、审计、统计、物价、人防、老龄、史志、档案、气象、残疾人事业等都有新发展。

七、人民生活有了新改善。农民人均纯收入达到3306元，城市居民家庭人均可支配收入达到7875 元，分别增长5.9%和6.1%。新增城镇就业和再就业3.16万人，城镇登记失业率控制在3.5%以内。社会保障体系进一步健全，“两个确保”、“一个低保”政策得到较好落实，在全省率先建立了农村特困群众救助制度。积极开展农村新型合作医疗试点工作，覆盖人口达到48万。通过改革经济适用房的实施办法，为城区572户中低收入家庭解决了住房困难，使当年享受经济适用房政策的户数接近前4年的总和，户均补助达到52447元，受到了社会各界的一致好评。通过实施农村饮水解困工程，为42个历史吃水困难村解决了人畜吃水问题，结束了农村人畜吃水困难的历史。农村基础设施建设得到加强，完成各类水利工程2115项，新增通柏油路村359个，实现了村村通客车，农村生产生活条件进一步改善。

八、政府自身建设呈现新气象。我们按照十五届人大一次会议上提出的“建设干事创业政府、一心为民政府、务实高效政府和公正廉洁政府”的要求，始终把干事创业、加快发展作为政府工作的第一要务。坚持开拓创新、团结实干，实现了发展上的高速度、高效率。日钢一期工程当年签约、当年开工、当年投产，创造了大项目建设史上前所未有的“日钢速度”。在与韩国平泽通航工作中，有关部门齐心协力，80天拆除建筑物4000多平方米、新建3000平方米候船大厅、1万多平方米的广场，以超常的节奏完成了各项通关准备工作，创造了令人称道的“通航速度”。在对外招商、园区拓展、市场化运作等经济和社会发展方面，都坚持以创新促创业、以创业促发展，全市上下呈现出锐意进取、生机勃勃的新气象。始终把公开、公平、公正作为政府工作的基本准则。我们以提高政府工作的透明度、扩大人民群众的知情权为目标，大力实施政府工作“阳光工程”。建立了重大事项新闻发布会制度；市政府全体会议邀请人大代表、政协委员、社会各界人士列席，并首次向全市直播；市政府常务会议邀请新闻媒体列席并公开报道；注重发挥舆论监督作用，在“抓双创、防非典”活动中，报纸、电台、电视台公开曝光783次，有力地推动了工作开展。积极推进民主科学决策，吸收各方面专家参加城市规划委员会，对重大规划项目实行专家论证和公示，对涉及群众切身利益的价格改革先听证后决策，既提高了决策的科学性，又从机制上探索了廉政建设的新路子。始终把执政为民作为政府工作的根本目的。认真组织实施五项“为民工程”，真心实意地为群众解难事、办实事、做好事，切实维护群众利益。特别是在防非典斗争中，各级领导干部勇于负责，靠前指挥，在夺取抗击非典胜利的同时，主动变困难为机遇，深入开展“抓双创、防非典”活动，实现了市民环境卫生意识明显提高、城区环境卫生质量明显改善、城市管理秩序明显变化“三个明显”的目标，赢得了全市人民的信任和支持。实践证明，只要我们心里装着群众，一切为了群众，紧紧依靠群众，我们的工作就一定能够得到人民群众的支持和拥护，我们的事业就一定能够兴旺发达！

总之，过去一年是我们经受非典疫情严峻考验、战胜各种困难、取得经济和社会发展新突破的一年，是全市广大干部群众满怀信心、大有作为的一年。经过全市上下的共同努力，我们实现了本届政府工作开好局、起好步的目标。日照市也在发展中出现了令人鼓舞的可喜变化：加快发展的信心足了。大家对日照的环境、港口、区位“三大优势”有了更加深刻的认识，亲身感受到了将潜在的自然优势转化为现实发展所带来的成功喜悦，极大地振奋了精神，鼓舞了士气，“别人做到的我们就能做到，别人做不到的我们经过努力也可以做得到”，已逐步成为全市上下的共识。有了这种敢打必胜的信心，我们就能战胜一切困难险阻，不断从胜利走向胜利！干事创业的氛围浓了。全市各级各部门心往一处想，劲往一处使，实现日照的跨越发展已成为全市人民的共同心愿。全市上下人心思上，人心思干，形成了干事创业、加快发展的强大合力和浓厚氛围，这是我们无比宝贵的巨大财富。有了这笔宝贵的财富，我们就能无往而不胜！思想观念变新了。用市场经济的手段抓发展、用发展的办法解决前进中的问题、用创新的精神培植发展新优势，正成为全市上下的自觉行动，各级干部驾驭市场经济的能力有了明显提高，社会各界的市场意识、开放意识、创新意识越来越强。只要我们坚持不懈地解放思想、更新观念、与时俱进，日照就能在重大战略机遇期中有所作为、赶超先进！对外形象变靓了。我市巨大的发展潜力和广阔的发展前景已被越来越多的人们所公认，同青岛、烟台、威海一样，日照正成为韩国产业转移的重点城市。只要我们抢抓机遇，努力把潜在的自然优势转化为现实的经济优势、发展优势、竞争优势，我们就一定能创造新的辉煌！

各位代表，过去一年我们所取得的这些成就，得益于党中央、国务院、省委、省政府和市委的正确领导，得益于市人大、市政协和社会各界的有效监督和大力支持，得益于历届市委、市政府打下的良好基础，是全市人民同心同德、干事创业的结果。在此，我代表市人民政府，向为日照改革开放和现代化建设做出贡献的全市人民，向所有关心、支持和帮助日照发展的同志们、朋友们，表示崇高的敬意和衷心的感谢！

过去一年取得的成绩令人鼓舞，但前进道路上还有不少困难和问题：日照经济欠发达的局面还没有根本改观，无论是经济总量还是人均占有，仍然低于全省平均水平；经济结构不合理的矛盾依然突出，产业结构、所有制结构、财政收入结构都存在着亟待解决的问题；实现农民增收的难度大，解决“三农”问题的任务艰巨；社会保障能力比较脆弱，就业和再就业压力加大；城市化水平不高，制约经济和社会发展的问题仍然存在；部分干部的思想观念、工作能力和水平还不能适应新时期政府工作的要求，官僚主义作风和腐败现象仍然存在；经济发展环境有待进一步改善。对这些困难和问题，我们将高度重视，采取得力措施加以解决。

2004年政府工作的任务

今年是实现“十五”计划的关键一年，也是全面落实十六届三中全会精神，深化改革、扩大开放、促进发展的重要一年。做好今年的政府工作，面临许多有利条件。一是我们仍处在加快发展的重要战略机遇期。国外产业向中国的转移、国际资本的涌入、国内民营资本扩张、扩大内需方针的继续实施，都会给经济社会发展带来巨大的机遇和活力。二是日照得天独厚的发展优势日益引起国内外的瞩目，对外吸引力越来越强，对我市经济社会发展的带动和促进作用越来越大。特别是一批临港工业大项目陆续落地建设并投产，为我们实现跨越发展创造了条件。三是经过全市上下的共同努力，我们已经在跨越发展的实践中理清了思路、积累了经验、探索了路子，锻炼了干部队伍，形成了人心思进、共谋发展的良好氛围。可以说，加快发展的天时、地利、人和俱在，环境、条件、基础并存，我们完全有能力、也一定能够实现日照的跨越发展！

今年全市经济和社会发展的总体要求是：以邓小平理论和“三个代表”重要思想为指导，深入贯彻党的十六大和十六届三中全会精神，牢固树立以人为本和全面、协调、可持续的发展观，坚持以加快发展为主题，以结构调整为主线，以改革开放为动力，继续实施港口立市、工业强市、科教兴市、生态建市“四大战略”，突出抓好招商引资、民营经济、特色经济、高新技术、农民增收“五项重点”，加快推进市场化、国际化、城市化“三化”进程，积极接轨青岛，奋力学赶威海，努力推动国民经济和社会事业跨越发展。主要预期目标是：国内生产总值增长16%；地方财政收入增长13%；新增就业3.2万人，城镇登记失业率控制在4%以内；城市居民家庭人均可支配收入增长8%，农民人均纯收入增长5%。今年政府工作的主要任务是：

一、高度重视“三农”问题，千方百计增加农民收入。把解决“三农”问题作为全局工作的重中之重，按照统筹城乡经济社会发展的要求，坚持“多予、少取、放活”的方针，认真落实各项扶持政策，促进农民增收、农业增效、农村经济社会全面发展。

继续推进农业和农村经济结构的战略性调整。在确保粮食安全的前提下，以茶、桑、果、菜、烟等高效经济作物为重点，进一步搞好种植业结构调整，大力发展优质高效农业、特色农业和创汇农业。继续推进农业内部结构调整，突出抓好以远洋捕捞、海珍品养殖和水产品精深加工为重点的海洋经济，全力加快以食草畜禽和规模养殖、加工为重点的畜牧业发展，努力提高水产业、畜牧业在农业中的比重。加快农村经济结构调整，大力发展农村二三产业，提高农民的非农收入。

加快农业产业化进程。把培植壮大农业龙头企业作为推进农业产业化的重中之重，加大扶持力度，落实优惠政策，努力增强辐射带动能力。紧紧抓住市场化取向、区域化布局、专业化种养、标准化生产四个关键环节，创新农业产业化经营机制，完善龙头企业与基地、农户的利益分配关系，保护农民利益，增加农民收入。加强农业社会化服务体系和农产品市场体系建设，健全农业科技推广网络，大力发展农产品交易市场，鼓励农村能人、基层涉农单位和龙头企业领办行业协会，发展农村合作经济组织，提高农民进入市场的组织化程度。

认真落实党在农村的各项政策。继续抓好《农村土地承包法》的贯彻实施，认真搞好“两田制”的清理整顿，稳定农村土地承包关系，严格耕地保护制度。继续深化农村税费改革和粮食流通体制改革，加强农村“两工”管理，切实减轻农民负担。除烟叶外全面取消农业特产税，同时降低农业税率1个百分点。放开粮食收购市场，改革粮食补贴办法。加强农业基础设施和生态环境建设，重点抓好百座水库除险加固、节水灌溉、小流域治理、沿海防潮堤和植树造林等工程，进一步改善农业生产条件。

加快农村劳动力转移，走通过减少农民富裕农民的新路子。把转移农村劳动力作为新时期解决“三农”问题、增加农民收入的关键环节来抓，认真落实省委、省政府《关于进一步做好农村劳动力转移工作的意见》，确保见到实效。实施农村劳动力转移“绿色通道”培训计划，健全完善市、县、乡、村四级劳务输出服务网络，提高外出务工农民的技能和组织化程度。加快户籍、用工和社会保障制度改革，消除影响劳动力转移的体制性障碍，改善转移就业环境。认真解决拖欠农民工工资等问题，依法保护进城务工农民的合法权益。年内实现农村劳动力转移2.3万人，其中组织跨区域就业1万人。

二、全面深化各项改革，加快完善市场经济体制。按照十六届三中全会《决定》要求，推动市场取向的改革向全方位、纵深化拓展延伸，为经济和社会发展增添活力。

大力培植多元化的市场主体。按照现代企业制度的要求，尽快完成公有企业改革任务，规范完善已改制企业，鼓励骨干企业与国内外大企业挂靠联合，推动有条件的企业上市融资。积极推进所有制结构调整，加快公有资本从一般竞争领域退出的步伐，鼓励民营企业、民间资本参与国有企业改革，大力发展混合所有制经济，逐步建立起归属清晰、权责明确、保护严格、流转顺畅的现代产权制度。进一步放宽市场准入，废除各种歧视性规定，革除一切思想障碍和体制障碍，放手发展非公有制经济，扶持优势民营企业做大做强。要适应市场经济发展的要求，大力培育多元化的市场主体，为加快发展奠定基础。

加快完善现代市场体系。按照统一、开放、竞争、有序的要求，加快土地、资本、技术、劳动力等要素市场的建设，积极发展中介服务机构，规范发展各类行业协会、商会等自律性组织，充分发挥市场对资源配置的基础性作用。深化金融体制改革，搞好农村信用社改革试点，建立和完善中小企业贷款担保等金融服务体系，加大金融对经济发展的支持力度。坚持行政执法、行业自律、舆论监督、群众参与相结合，进一步整顿和规范市场经济秩序，建立政府、企业和个人社会信用体系，加快“诚信日照”建设。

积极推进事业单位改革。加大事业单位管理体制、用人制度、分配制度改革力度，加快经营性事业单位事改企、企改制的步伐，尽快建立适应市场经济要求的运行机制。进一步放开城市基础设施、公用事业建设经营市场，逐步建立起投资多元化、经营企业化、服务规范化、管理法制化的城市公用事业建管体系。积极稳妥地推进科、教、文、卫、体等其他事业的改革，一方面加大对基础教育、公益性文化、竞技体育、公共卫生事业的投入，另一方面要深化市场取向的改革，积极探索产业化发展的新路子。

切实转变政府职能。深化行政管理体制改革，认真解决政府管理越位、缺位和错位问题，政府该管的要坚决管好，不该管的坚决放开，能用市场手段解决的决不用行政手段，能交给中介机构办的坚决交给中介机构去办，实现政府职能由无限向有限、由管理型向管理服务型的转变。转变行政理念，自觉运用经济和法律手段管理经济和社会事务，今后政府采购的不单是办公用品，还要采购设计、采购工程、采购管理等等。继续深化行政审批制度改革，强化行政服务中心的职能，促进政府工作提速提效。按照转变政府职能的要求，协调推进部门预算、国库集中收付、政府采购和收支两条线管理的改革，尽快形成公共财政基本框架。全面落实各项税收改革措施，大力推进社会综合治税，充分发挥财税政策在促进经济增长、优化结构和调节收入方面的重要作用。

三、加快经济国际化进程，全面提高对外开放水平。坚持以开放促改革、促发展、促调整，在更大范围、更广领域和更高层次上参与国际合作与竞争。

突出抓好招商引资。紧紧抓住国际产业转移加快的历史机遇，进一步加大争引力度，确保今年实际利用外资再翻一番。在招商对象上突出韩日台港，坚持“走出去”与“请进来”相结合，广泛开展各类招商活动，努力做好“借韩兴市”的文章。在招商领域上突出争引工业项目，加快旅游业、服务业、城市建设和农业领域的利用外资。在招商方式上突出专业招商、以商招商等形式，增强招商引资的实效性。在招商载体上突出园区和现有骨干企业两个重点，着力抓好现有园区的整合与发展，提高园区的集约化水平。同时，强化日照以外都是外、外币本币都是钱、内资外资一齐引的观念，进一步加大国内招商力度，积极引导全国各地的各类企业、各种大进大出的产业向我市转移。

加快构筑对外经贸新格局。把加工贸易作为发展外向型经济的突破口，积极争取建立国家级出口加工区，多方承接和兴办加工贸易项目，尽快把我市建成周边国家和地区的加工基地、装配基地、出口基地。继续实施市场多元化战略，全方位、多层次开拓国际市场，落实好中央出口退税改革政策，千方百计扩大外贸出口，全年进出口总值增长25%。积极实施“走出去”战略，鼓励有条件的企业到国外投资办厂、开展工程承包和劳务输出，促进对外经济的快速增长。

进一步改善投资环境。牢固树立环境是资源、是竞争力的意识，变先发展经济后改善环境为先改善环境，努力创造一流的服务环境、政策环境、法制环境、市场环境。加快思想观念和行为方式与国际惯例接轨步伐，增强日照对外来投资者的吸引力和招商引资的成功率，营造亲商、富商、安商的浓厚氛围。

四、积极实施“工业强市”战略，全力加快制造业发展。紧紧抓住省委、省政府建设半岛制造业基地的机遇，充分发挥我市邻近日韩、交通便利的优势，积极承接外来产业转移，不断壮大工业实力，加快形成工业主导型的经济增长格局。

大力发展临港工业。依托港口优势，加大对冶金、粮油加工、液体化工、木制品加工、浆纸、能源六大临港支柱产业的培育力度，推动上下游企业纵向组合，拉长产业链条。按照“投产一批、开工一批、储备一批”的要求，抓好重点工业项目建设，加快日钢二期、济钢一期工程建设，尽快启动日照电厂二期和100万吨木浆项目的建设，积极推进沥青加工等项目前期工作，全力把临港工业的规模做大、产业链做长，把临港企业做大做强，使之能成为省内乃至国内同行业的排头兵，努力形成富有日照特色的产业集群。

加快改造提升传统产业。坚持以膨胀规模、优化结构、提高效益为主攻方向，积极运用先进适用技术、高新技术和信息化手段，搞好机械、食品、建材、轻纺等传统产业的改造，加快培育优势产业和骨干企业。加大技术改造投入，继续实施百项重点工业项目，技改投资要增长40%以上。加大对森博、五征等35户重点企业的扶持力度，着力培育优势产业、强势集团和知名品牌。鼓励优势企业以资本为纽带，实行跨地区、跨行业、跨所有制联合，做大做强现有企业，力争年内有7家企业销售收入过10亿元，其中1家企业过40亿元；有40家企业实现利税过千万元，其中3家企业过亿元。

积极发展高新技术产业。围绕生物技术、电子信息、新材料三大领域，多渠道增加投入，加快培植高新技术产业，形成新的工业经济增长点。充分发挥大学科技园、北大产学研居基地等人才聚集的优势，做好科研成果转化的文章。搞好优力凯生物工程研究中心、鼎弘电子玻璃等项目建设，力争省级高新技术企业超过20家，高新技术产业产值达到20亿元以上。

五、加快发展特色经济，繁荣城市服务业。充分发挥我市港路、区位、生态环境优势，大力发展新型特色经济和现代服务业，打造地方经济特色品牌。

大力发展港口经济。把港口经济作为我市特色经济的主体来抓，认真实施《港口法》，加强岸线资源的科学规划和控制，鼓励国内外经济组织和个人依法投资建设、经营港口，全力加快港口发展。积极推进日照港矿石码头、集装箱码头等十大工程建设，打通日照港东西港区，启动岚山港北港区和日照中心渔港建设，修筑日照港至岚山港高速通道。依托深水大港的优势，加快培育六大临港工业，努力实现港口建设和临港工业互相促进、共同发展。加快培育以港口物流为核心的现代物流业，抓好日照港焦炭物流园建设，年内完成港口货物吞吐量5500万吨，集装箱运量12万标箱，海上国际客运量超过5万人。

发展壮大旅游经济。充分利用好中国优秀旅游城市这块金字招牌，坚持“吃、住、行、游、购、娱”综合开发，培育壮大旅游支柱产业。加快景区景点开发建设，重点抓好太阳城主题公园、海洋生物馆、太公岛游乐园等一批旅游项目，搞好与“山东黄金海岸游”和“山水圣人游”两条旅游线的衔接，积极发展海滨度假旅游，精心打造日照的旅游品牌。完善旅游配套设施，抓好旅游商品、纪念品和特色餐饮的开发，提高旅游业综合效益。争取日照至西安旅游专列旺季对开，扩大旅游客源市场。加大旅游环境整治力度，规范旅游市场秩序，树立海滨旅游城市的良好形象。

做大做强房地产业。房地产业素有永远的朝阳产业之称，我市地处沿海，生态环境优良，房地产市场潜力巨大，发展前景广阔。要创造公平竞争的环境，规范房地产市场秩序。全面放开已购公房上市交易，简化手续，降低收费，促进住房二级市场的繁荣。加大对日照优良的生态环境和人居环境的宣传力度，进一步提高日照房地产的知名度，拉动房地产业快速发展。

积极培育大学经济。坚持高等教育与科技研发并重，努力把大学科技园建成高等教育基地、科技创新基地和成果转化基地。开工建设山东体育学院三期和西安翻译学院、中国地质大学海洋学院一期工程，完成济宁医学院、山东水利职业学院、日照职业技术学院一期工程，新引进大学2所，在校大学生达到4万人。

探索发展会展经济。立足日照优美的自然环境，搞好会展设施建设，扩大对外宣传，加快培育日照的会展品牌。突出抓好世帆赛设施、市会展中心等场馆的建设，提高承接大型会展、赛事活动的能力。继续搞好茶博会、房博会等大型会展活动，全力做好2005年和2006年世帆赛筹备工作，办好2004年全国帆船锦标赛，提升日照城市形象。

加快发展现代服务业。突出连锁经营、物流配送、批发市场三个重点，加快流通现代化建设，年内完成现代物流和城市商业网点规划，加快推进南方家园物流商城、温州物流工业商城、中国日用商品城等物流项目建设，搞好银座商城、利群商厦、凯旋广场等大型商业设施建设。积极培育信息产业，加快推进电子政务、电子商务建设，以信息化带动工业化、城市化。

六、坚持科学的发展观，促进协调发展。牢固树立全面、协调、可持续的发展观，是做好政府工作必须长期坚持的重要指导思想，也是解决当前诸多矛盾必须遵循的基本原则。

统筹城乡发展，加快城市化进程。进一步提高城市规划、建设、管理水平，抓好投资亿元以上的帆船赛港池建设和灯塔广场、火车站广场改造等重点工程，建成垃圾处理厂。继续实施城市环境综合整治和绿化、美化、亮化工程，新增绿化面积260万平方米。全力提高城市的繁荣程度，大力发展高档次大型商场、星级宾馆及高雅文化娱乐设施，努力让日照美起来、亮起来、繁华起来。进一步完善城市管理体制，搞好城市社区建设试点。加强中心镇驻地和农村小城镇建设，鼓励镇村经济实体向小城镇集中，加快农村城镇化步伐，推动城乡一体化发展。

统筹区域发展，培育壮大县域经济。把培育壮大县域经济作为增强全市经济综合实力的重大举措来抓，加大招商引资力度，突出工业强县这个重点，大力发展特色经济、民营经济，加快县域经济结构调整。积极推进县域综合改革，增强发展活力。要努力为县域经济的发展创造条件，加大政策扶持力度，加快市区至岚山、市区至五莲县潮河镇的公路建设，为县域经济发展创造良好环境。

统筹经济社会发展，促进全面进步。要深化科技体制改革，提高科技创新能力，加快科技进步。牢固树立人才资源是第一资源的观念，大力培养和引进各类人才，创新人才工作机制，为跨越发展提供人才智力保障。巩固提高“普九”成果，大力发展高等教育、职业教育、成人教育和民办教育，构建终身教育体系。广泛开展群众性文体活动，加快广电事业发展，努力培植文化产业。要高度重视人民的生命健康，努力提高城乡卫生医疗服务水平。年内投资1000万元建设市疾病控制中心和传染病医院，提高公共卫生应急处置能力。认真落实《中医药条例》，重视中医中药的发展。

统筹人与自然和谐发展，切实做好人口资源环境工作。继续稳定低生育水平，全面推进计划生育优质服务，提高出生人口素质。依法加强土地、矿产、海洋和水资源管理，实现各类资源可持续利用。妥善处理加快发展与环境保护的关系，大力推进生态市建设，加大环境保护的力度，编制并实施《循环经济发展规划》。要全力做好国家卫生城市和国家环保模范城市创建工作，努力把日照建设成为经济繁荣、环境优美、社会文明的生态城市。七、关心群众生产生活，认真解决关系群众切身利益的问题。进一步增强群众观念，紧紧围绕人民群众最关心的生产、生活问题，搞好“民心工程”建设，诚心诚意地为群众解难事、办实事、做好事，真正成为人民群众的知心人、贴心人。

千方百计做好就业和再就业工作。以对人民群众高度负责的精神，把扩大就业摆在更加突出的位置，全面落实扶持就业和再就业的各项政策。把增加就业的立足点放在加快发展上，大力发展服务业、劳动密集型企业和劳务输出。鼓励支持下岗失业人员自主创业、自谋职业、灵活就业。加大就业再就业投入，完善公共就业服务制度和再就业援助制度，积极开发公益性岗位，拓宽就业渠道。

加快建立与经济发展水平相适应的社会保障体系。以扩面征缴为重点，健全完善养老、失业、医疗、工伤、生育等社会保险制度，积极推进企业退休人员社会化管理服务工作。切实抓好城市居民最低生活保障、农村特困群众救助、社会救济、社会福利和慈善事业，加大对弱势群体的救助力度。全面推广新型农村合作医疗制度，确保农民群众“小病看得起、大病不返贫”。巩固扩大经济适用住房改革成果，使今年享受经济适用房补贴的户数仍不少于500户。

妥善解决好群众关心的问题。引导各级干部像重视财政收入那样重视农民增收，多为农民增收出主意、想办法，做到在任何时候、任何情况下都不增加农民负担，都不损害农民利益。抓好农村人畜饮水改善工程，年内完成80个村饮水改善任务。加快推进村村通柏油路工程，完成农村公路改造930公里。进一步完善社会救助制度，安排好灾区和困难群众的生产生活。

八、加强政治文明和精神文明建设，推进民主法制进程。坚持用邓小平理论和“三个代表”重要思想武装头脑，指导实践，推动工作。全面落实《公民道德实施纲要》，广泛开展精神文明创建活动，积极推进城市文化建设，不断提高人们的思想道德水平和文明程度。加强全民国防教育，落实优抚安置政策，增进军政军民团结，投资1500万元建设民兵预备役训练基地。加强社会主义民主法制建设，各级政府要自觉接受人大及其常委会的法律监督、工作监督和政协的民主监督，主动加强与各民主党派、人民团体、无党派爱国人士的联系，广泛听取各方面的意见和建议，实行科学民主决策。加强基层民主政治建设，推行政务公开、厂务公开、村务公开。全面实施“四五”普法和第二个依法治市五年规划，积极开展法律援助，提高依法治理水平。加强社会治安综合治理，严厉打击各种犯罪，继续开展同“法轮功”等邪教组织的斗争，妥善处理人民内部矛盾，努力创建“平安日照”。严格落实安全生产责任制和事故责任追究制，坚决杜绝各类重大安全责任事故的发生，确保人民生命财产的安全。

切实加强政府自身建设

实现日照的跨越发展，各级政府重任在肩，责无旁贷。我们一定按照胡锦涛总书记提出的“为民、务实、清廉”的要求，继续履行“建设干事创业政府，做到创业有为；建设一心为民政府，做到为民有绩；建设务实高效政府，做到工作有效；建设公正廉洁政府，做到廉政有方”的承诺，不断加强政府自身建设，全力开创政府工作的新局面。

一、坚持始终把加快发展作为政府工作的第一要务。继续发扬政府工作的团队精神、敬业精神、务实精神、创新精神和延安精神等“五种精神”，进一步增强忧患意识、超前意识、机遇意识和做一流工作、创一流业绩的意识等“四种意识”，真正做到聚精会神搞建设，一心一意谋发展。坚持以干事创业来论工作、评干部，旗帜鲜明地支持鼓励创业者、关心爱护改革者、批评教育空谈者、追究惩治诬陷者，努力营造干事创业的良好氛围，保护好各方面干事创业的积极性，用共同的事业凝聚人、激励人。要不断提高工作本领，增强创业能力，善于做好上级指示精神与本地实际相结合的文章，做到不仅想干事、能干事，而且会干事、能干成事。要在加快发展中树立新形象、展现新作为、创造新业绩，努力把政府建设成为能带领全市人民艰苦创业的“责任政府”！

二、坚持始终把为民谋利作为政府工作的根本出发点和落脚点。时刻把群众的冷暖疾苦挂在心上，满腔热情地为群众解难事、办实事，切实维护好、实现好、发展好最广大人民群众的根本利益，让全市人民从改革发展中得到更多的实惠。特别要高度重视和解决好农民增收、城市就业、社会保障、弱势群体救助等与群众切身利益息息相关的问题，真正做到权为民所用、情为民所系、利为民所谋。坚持走群众路线，把群众的呼声作为第一信号，政府一切工作都要以“群众拥护不拥护、赞成不赞成、高兴不高兴、答应不答应”为最高标准，努力把政府建设成为顺民心、合民意的政府，建设成为能赢得人民群众信任、理解和支持的“民心政府”！

三、坚持把提高政府执政能力和水平作为干部队伍建设的重点紧紧抓住不放。各级政府公务员特别是领导干部要着力提高三个能力：一是提高依法行政的能力。把学习贯彻实施《行政许可法》作为政府法制建设的一项重大而紧迫的任务，加强对行政执法人员的培训，认真搞好行政执法内容和程序的规范，强化行政监督，切实做到有权必有责、用权受监督、侵权要赔偿。二是提高驾驭市场经济的能力。充分认识市场在资源配置中的基础性作用，树立与市场经济要求相适应的思想观念和思维方式，学会按市场经济规律和国际规则办事，用经济的手段解决发展中的问题，提高面向市场、运用市场抓发展的能力。三是提高总揽全局、处理复杂问题的能力。各级政府和各部门的负责同志要实行“一岗双责”，一手抓经济发展，一手抓社会稳定，妥善处理好各类社会矛盾，特别是新时期的人民内部矛盾，切实担当起富一方百姓、保一方平安的政治责任。要在政府工作中大力弘扬“实事求是”的精神和务实高效的作风，坚持说实话、办实事、求实效，坚持立说立行、真抓实干。要树立正确的政绩观，坚决克服形式主义和官僚主义，坚决反对光说不做、弄虚作假、做表面文章的行为，坚决禁止搞任何形式的“政绩工程”、“形象工程”！

四、坚持把提高政府工作透明度、扩大群众知情权作为政府廉政建设的重点切实抓紧抓好。大力实施政府工作“阳光工程”，完善人民建议征集、重大事项社会公示、社会听证、专家咨询等制度，凡是政府重大决策、重大突发事件的处置、重大基建项目招投标和政府投资兴建的社会公益事业等情况，都要及时向社会公开，自觉接受社会各界的监督，坚决杜绝暗箱操作、权钱交易行为，从源头上预防和遏制腐败现象的发生。注重发挥舆论监督的作用，采用“焦点访谈”的形式，对政府工作人员履行公务情况进行明察暗访，严肃查处各种违法违纪行为。全面落实廉政建设责任制和廉政准则，切实纠正部门和行业不正之风，深入开展反腐败斗争，坚决清除腐败分子，努力把政府建设成为清正廉洁、公开、公平、公正的“阳光政府”！

各位代表！

日照是一个有着巨大发展潜力和广阔发展前景的城市，实现日照经济的跨越发展，是历史赋予我们的庄严使命，是全市人民的期盼。尽管前进道路上会有各种困难和挑战，但任何困难和挑战都动摇不了日照人民加快发展的决心！日照人民已经创造了令人自豪的历史，也一定能够创造更加辉煌的未来！让我们紧密团结在以胡锦涛同志为总书记的党中央周围，高举邓小平理论伟大旗帜，全面贯彻“三个代表”重要思想，在中共日照市委的正确领导下，解放思想，与时俱进，同心同德，开拓进取，为实现跨越发展的宏伟目标而努力奋斗！
